# Importance of Studying Non-Coding RNA in Children and Adolescents with Type 1 Diabetes

**DOI:** 10.3390/biomedicines12091988

**Published:** 2024-09-02

**Authors:** Manuela Cabiati, Giovanni Federico, Silvia Del Ry

**Affiliations:** 1 Laboratory of Biochemistry and Molecular Biology, Institute of Clinical Physiology, National Research Council (CNR), 56124 Pisa, Italy; manuela.cabiati@cnr.it; 2 Department of Clinical and Experimental Medicine, University of Pisa, 56126 Pisa, Italy; giovanni.federico@med.unipi.it

**Keywords:** type 1 diabetes (T1D), children, biomarkers, miRNA, long non-coding RNA, circular RNA

## Abstract

Type 1 diabetes (T1D) mellitus is a chronic illness in children and teens, with rising global incidence rates. It stems from an autoimmune attack on pancreatic β cells, leading to insufficient insulin production. Genetic susceptibility and environmental triggers initiate this process. Early detection is possible by identifying multiple autoantibodies, which aids in predicting future T1D development. A new staging system highlights T1D’s onset with islet autoimmunity rather than symptoms. Family members of T1D patients face a significantly increased risk of T1D. Italy recently passed a law mandating national T1D screening for pediatric populations. Measurements of β cell function continue to be essential in assessing efficacy, and different models have been proposed, but more appropriate biomarkers are mandatory for both progression studies before the onset of diabetes and during therapeutic monitoring. Biomarkers like microRNAs (miRNAs), long non-coding RNAs (lncRNAs), and circular RNAs (circRNAs) play key roles in T1D pathogenesis by regulating gene expression. Understanding their roles offers insights into T1D mechanisms and potential therapeutic targets. In this review, we summarized recent progress in the roles of some non-coding RNAs (ncRNAs) in the pathogenesis of T1D, with particular attention to miRNAs, lncRNAs, and circRNAs.

## 1. Introduction

Type 1 diabetes (T1D) mellitus stands as a prevalent chronic ailment among the young, particularly in children and adolescents, affecting 1 in 400 [1], and there is evidence that the frequency of childhood T1D is increasing worldwide [2], with reported increases of 2 to 5 percent per year in Europe, the Middle East, and Australia. From the last IDF report in 2021 [3], the number of children and adolescents with T1D worldwide is 1,211,900 (54% being <15 years), and the number of newly diagnosed cases each year is 108,500–149,500 (<20 years). Europe has the highest number of children with T1D—approximately 300,000—compared with the other IDF regions. Europe also has one of the main incidence rates of T1D in children, with an estimate of about 30,000 new cases per year [4]. T1D arises from an autoimmune response where the body’s immune system targets and damages the insulin-producing beta cells in the pancreas. Consequently, insulin production diminishes significantly or ceases altogether. The precise triggers for this immune-mediated assault remain incompletely elucidated; however, it is probable that a blend of genetic predisposition, influenced by numerous genes, coupled with an environmental catalyst like a viral infection, initiates the autoimmune response [5,6].

George Eisenbarth’s characterization of T1D as a chronic autoimmune condition, depicted by autoimmunity and a gradual decline in beta cell function until there is insufficient beta cell mass to regulate symptomatic hyperglycemia, has long served as the cornerstone of T1D’s natural history paradigm [7]. While the “Eisenbarth” model has evolved and recognized that autoimmunity and beta cell dysfunction precede diagnosis, it acknowledges the stepwise and non-linear nature of these changes. Additionally, it acknowledges the possibility that beta cell destruction may not be complete. As reported in the ISPAD Clinical Practice Consensus Guidelines 2022 [8], T1D is, in fact, characterized by four stages: Stage 1 is identified by the presence of multiple islet autoantibodies, normal blood glucose, and no clinical symptoms; Stage 2 has multiple islet autoantibodies, abnormal glucose tolerance, and usually no symptoms; Stage 3 has multiple islet autoantibodies, blood glucose above ADA diagnostic thresholds, and presence of clinical symptoms; and Stage 4 is identified by the presence of established, long-standing T1D. This paradigm, stemming from decades of collaborative research involving numerous investigators and the active involvement of thousands of T1D patients and their families, forms the fundamental basis for T1D clinical trials.

Differentiation and proliferation of autoreactive T cells (CD4+ T) is key in T1D. Naïve CD4+ T cells can differentiate into T helper (Th) cells under stimulation by different cytokines. Biased differentiation of naïve CD4+ T into Th1 in the early stages contributes to T1D development; subsequently, Th17 is induced to maintain the progression of T1D. Impaired differentiation or survival of Th2 and nTregs has also been attributed to this pathological process. It eventually activates cytotoxic T lymphocytes (CTLs) to attack beta cells. The significance of different CD4+ T subgroups in T1D development has been reported [9,10,11,12].

Recent studies indicate that in some types of autoimmunity, the interaction between the environment and the host is influenced by epigenetic alterations induced by many environmental aspects, including altered DNA methylation patterns [13]. Environmental influences can disrupt the maintenance of epigenetic stability in certain cells, potentially leading to a loss of tolerance through aberrant gene expression. While epigenetic alterations affect gene expression and cellular processes, the underlying genomic sequence remains unaltered. Key epigenetic mechanisms involve non-coding RNA expression, modification of amino termini of histone proteins by post-translational modifications, and methylation and/or hydroxymethylation at CpG dinucleotides [14].

Understanding how genetic and epigenetic pathways intersect contributes to our comprehension of autoimmune diseases. A newly identified group of non-coding RNA (ncRNA) plays a critical role in regulating both autoimmune and immunological processes.

Despite advancements in medicine, the treatment landscape for autoimmune diseases has seen minimal evolution over recent decades, with many disease mechanisms remaining elusive. Comprehending the initiation, progression, and resolution of autoimmune ailments is paramount. Given it distinct regulatory attributes and pathological implications, ncRNA emerges as promising biomarker candidates for accurately diagnosing the progression of autoimmune diseases [15,16,17].

Non-coding RNAs are a group of RNA molecules that, while not encoding proteins, play significant roles in regulating gene expression at the post-transcriptional level and in epigenetic gene silencing. Primarily derived from introns found in both protein-coding and non-coding genes [18], ncRNAs encompass diverse categories such as miRNAs, lncRNAs, circRNAs, siRNAs, ceRNAs, and piRNAs [19].

**Figure 1:**
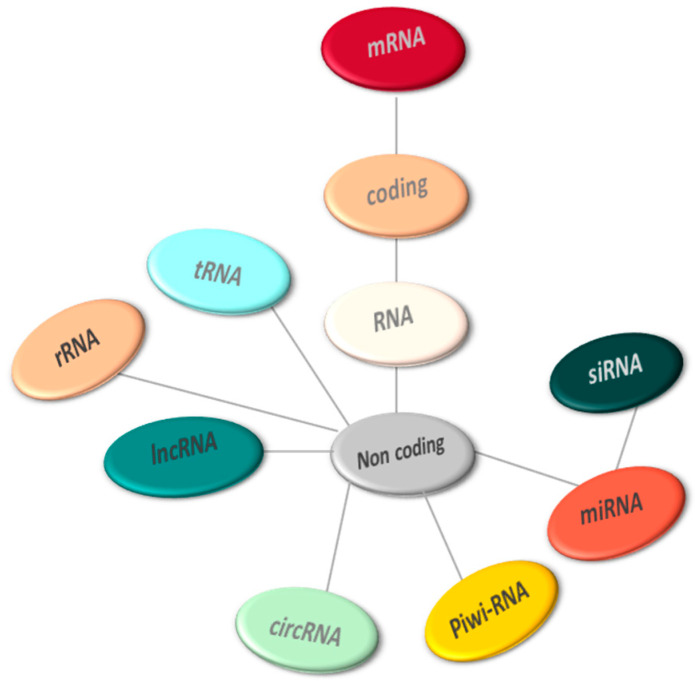
Members of the non-coding RNA world modified from Ref. [20].

They can be subdivided, based on size, into two major groups: (1) Small ncRNAs (<200 nucleotides long), which include miRNAs, PIWI-interacting RNAs, and endogenous short interfering RNAs; (2) lncRNAs, which have a length between 0.2 and 2 kb [20,21] (Figure 1).

MiRNAs, a subset of small endogenous molecules, typically around 21 nucleotides long, play crucial roles in regulating cell cycle progression, differentiation, angiogenesis, and apoptosis [22]. Studies suggest that miRNAs possess complementarity to their target genes, enabling them to regulate various mRNA targets [23]. MiRNAs primarily influence the post-transcriptional regulation of gene expression by either inhibiting mRNA translation or promoting mRNA degradation. Primary miRNA (pri-miRNA) transcripts, which contain hairpins and 5′ and 3′ flanking sequences, are produced by RNA polymerase II. The processing of these transcripts is mainly conducted by Drosha and Dicer, two enzymes from the RNase III family. These enzymes function in complexes with dsRNA-binding proteins (dsRBPs), specifically DGCR8 and transactivation-responsive RNA-binding protein (TRBP) in mammals, to facilitate the two steps of primary precursor (pre-miRNA) processing in the canonical pathway. The structural properties of individual pri-miRNA sequences influence the efficiency of pri-miRNA processing. Co-transcriptional processing of pri-miRNAs leads to a rapid formation of 59–71 nucleotide-long stem-loop pre-miRNAs. Exportin-5, a member of the karyopherin protein family, transports nascent pre-miRNAs to the cytoplasm in a GTP-dependent manner. Once in the cytoplasm, pre-miRNAs are processed by the RISC loading complex (RLC) and converted into 21-nucleotide-long miRNA/miRNA* duplexes by the type III enzyme ribonuclease Dicer. It is estimated that up to one-third of human mRNAs may be miRNA targets. miRNA-mediated gene regulation is crucial for normal physiological processes, including cell cycle differentiation and cell death [24,25]. Current research highlights the importance of miRNAs in regulating immunological processes and in avoiding autoimmune diseases [17,26].

Long non-coding RNAs do not have a known protein-coding function. However, this definition may be preliminary: indeed, many lncRNAs have recently been reported to encode short peptides in human tissues. Nonetheless, lncRNAs can be categorized as follows: (a) Sense lncRNAs, which overlap one or more exons of another transcript on the same strand; (b) Antisense lncRNAs, which overlap one or more exons of another transcript on the opposite strand; (c) Bidirectional lncRNAs, whose expression and that of a neighboring coding transcript on the opposite strand are initiated in close genomic proximity; (d) Intronic lncRNAs, derived from an intron of a second transcript; (e) Intergenic lncRNAs, found as an independent unit within the genomic interval between two genes. The precise mechanisms of the various functions of lncRNAs are under intense investigation. They can interact with a wide range of macromolecules within the cell, including other RNA species, proteins, and DNA. Complementary sites on lncRNAs enable them to recognize and bind to mRNAs, microRNAs, or even other lncRNAs, acting as highly specific sensors for their regulation. Protein-binding sites allow interactions with proteins, forming ribonucleoprotein particles with diverse functions. The formation of binding sites and the ability to undergo allosteric transitions are possible due to the capacity of these ncRNAs to fold into various thermodynamically stable secondary structures, generating a complex structural scenery. Unlike mRNAs, the lncRNAs possess high folding energy [27,28]. In general, lncRNAs can be classified into nuclear and cytoplasmic categories: nuclear lncRNAs guide chromatin modifiers such as DNA or histone methyltransferases and polycomb repressive complex 2 to specific genomic loci, resulting in the induction of a repressive heterochromatin state and, thus, transcriptional downregulation. Cytoplasmic lncRNAs modulate gene expression positively/negatively at the translational level by binding to targeted mRNAs or acting as competing endogenous RNAs that sequester microRNAs, preventing them from exerting translational repression on their targets [29].

Investigating ncRNAs in T1D, obtained from cellular multivesicular bodies enriched in specific miRNAs, is extremely important as they may play a significant role as biomarkers to assess disease progression and potentially provide a disease-specific diagnostic signature [30]. Exosomes (EXOs) are small lipid vesicles, approximately 30–200 nm in diameter, derived from multivesicular bodies (EV) and released by closely all cell types. They have emerged as crucial mediators of cell communication, transferring proteins, lipids, and RNA species (including miRNA, mRNA, and tRNAs) between cells. Additionally, EXOs are often enriched with a subset of miRNAs present in the parent cell. They can be found in various body fluids such as serum, urine, cerebrospinal fluid, saliva, and bronchoalveolar lavage fluid. The RNA content of EXOs from different sources can reflect biological events and disease processes. Specifically, profiling miRNAs in circulating exosomes has shown potential for diagnosing various diseases. Plasma-derived exosomes enriched with specific miRNAs could provide a disease-specific diagnostic signature, which supports the prediction and monitoring of T1D [31,32], and it was demonstrated that miRNAs found in exosomes isolated from the plasma of T1D subjects could serve as a potential diagnostic biomarker [30]. Recent studies have also highlighted that certain miRNAs contribute to the progression of diabetes mellitus and to the potential complications related to cardiovascular diseases that can arise in diabetic patients. An example can be the miR-29 family members that are regulated in a concerted fashion in multiple tissues, including the heart and pancreas, and are early markers of diabetes mellitus. They are downregulated under hyperinsulinemic conditions but increase dramatically in response to loss of hyperinsulinemia and elevated plasma glucose levels [33]. To date, many studies revealed that exosomal miRNAs (such as miRNA-125b, miRNA-144, let-7, miRNA-155, miRNA-29, miRNA-133a, and miRNA-7) promote the development and progression of diabetes [34]. In addition, ncRNAs such as lncRNA PVT1, LINC00960, and hsa-miR-107 might be involved in inflammatory response in T1D, serving as novel biomarkers and potential targets for the diagnosis and treatment of T1D [35].

Recent examinations highlight the critical role of ncRNAs, in particular miRNAs, lncRNA, and circRNAs, in governing immunological processes and their pivotal function in averting autoimmune disorders [15,16,17].

However, the great challenge remains how to identify children at high risk of developing diabetes early.

The presence of two or more autoantibodies targeting islet antigens such as insulin, glutamic acid decarboxylase (GAD65), protein tyrosine phosphatase-like islet antigen 2 (IA2), or zinc transporter 8 protein (ZnT8) in early childhood indicates a heightened risk of future T1D development. Diabetes-specific autoantibodies may manifest years before clinical diagnosis, offering a reliable means of predicting disease progression, and it has been reported that family members of a person with T1D are at increased risk, 15 folds higher than the general population, to develop T1D [36].

Thus, to date, risk assessment utilizing autoantibodies has relied on the number of autoantibodies present, with compelling evidence indicating that nearly all individuals positive for multiple autoantibodies will eventually develop clinical disease. Nonetheless, longitudinal investigations have revealed significant variability in the progression rate among those positive for multiple autoantibodies. Precise identification of individuals with the swiftest disease progression is vital for effective and informative clinical trials, as well as for pinpointing candidates most likely to benefit from interventions aimed at modifying the disease course [37]. Recently, in fact, the FDA approved an anti-CD3 antibody, Teplizumab, able to delay progression to clinical T1D when given to children and adolescents who did not have diabetes but were at high risk for the development of clinical disease; they were in stage 2 of T1D progression. In these subjects, as compared with the placebo group, the median delay in the diagnosis of diabetes was 2 years after a 2-week course of treatment with teplizumab [38]. Teplizumab administration, as compared with placebo, was also effective in delaying the progression of beta cell failure, measured as stimulated C-peptide secretion, in children and adolescents with newly diagnosed T1D [39]. In addition, it was reported that, compared with placebo, verapamil, a well-known calcium channel blocker, partially preserved stimulated C-peptide secretion at 52 weeks from diagnosis in children and adolescents with newly diagnosed T1D [40].

Another important consideration is assessing the risk for the development of clinical diabetes is essential to reduce the likelihood of experiencing diabetic ketoacidosis (DKA), a serious, life-threatening acute complication of T1D [41] occurring when tissues fail to utilize simple sugar (glucose) as an energy reserve because there is either an absolute or even partial deficiency of insulin production.

Predicting overt T1D has been considered a very important issue in Italy, and the Italian Parliament approved, very recently, a law establishing a national screening for T1D (recognition of islet-specific autoantibodies) to be offered to the pediatric population (Law 15 September 2023, n. 130: GU n.226 del 27-9-2023).

While islet autoantibodies play a pivotal role in risk assessment, they may not be the most effective biomarkers for monitoring efficacy. Clinical trials focusing on monitoring islet autoantibodies have yielded limited results. Evaluating beta cell function remains crucial for assessing efficacy, and various models have been proposed for this purpose. However, there is a pressing need for more suitable biomarkers for both progression studies, conducted before diabetes onset, and therapeutic monitoring. A recent review [42] underscored the significance of biomarker development for monitoring the rate of T1DM progression and therapeutic response. It highlighted non-coding RNA (ncRNA) as an emerging area worthy of further exploration in T1D research.

This review provides an overview of recent advancements in understanding the roles of various ncRNAs, particularly miRNAs, lncRNAs, and circRNAs, in the pathogenesis of T1D.

### 1.1. miRNAs and T1D

As mentioned above, there is a need for new biomarkers to complement the information gathered from autoantibodies, genetic predisposition, and environmental factors in the characterization of children and adolescents with diabetes [43], and several miRNAs have emerged as potential biomarkers for assessing health status and disease progression [44]. It was estimated that miRNAs regulate the expression of more than 60% of protein-coding genes [45,46,47].

MiRNAs can be readily isolated from various biological specimens and exhibit durability, owing to their protection by microsomes and exosomes, which form a shielding outer layer for the miRNAs.

Numerous studies have identified a plethora of miRNAs showing differential expression in samples from individuals with T1D [44,46]. These investigations have been conducted using cultured cells, body fluids, or solid tissue samples from T1D patients or murine models of the disease, employing various techniques to quantify gene expression. However, the findings across studies exhibit inconsistency, with only a handful of miRNAs identified thus far as significant signatures of T1D.

One such specific miRNA is miR-21, which has been demonstrated to impede beta cell development in animal models of T1D when overexpressed [48]. Additionally, miR-21 targets the translation of the Bcl-2 gene, leading to heightened beta cell apoptosis during diabetes progression [49,50].

In previous experimental models of T1D, studies have shown that beta cell miR-21-5p levels rise in response to treatment with inflammatory cytokines, leading to detrimental effects on beta cell survival and function [49,51,52,53,54]. Notably, this increase in miR-21-5p appears to be specific to inflammation, as exposure to high glucose to simulate hyperglycemia or tunicamycin to induce endoplasmic reticulum stress does not affect miR-21-5p expression [51]. Multiple research groups have reported elevated circulating levels of miR-21-5p in individuals with long-standing T1D [55,56]. However, the underlying cause of these observed increases in circulating miR-21-5p and its potential as a biomarker for developing T1D remain unexplored. Promising studies have investigated whether exposure to the microenvironment of developing T1D would lead to increased beta cell EVs miR-21-5p and whether circulating EV miR-21-5p could serve as a biomarker for this process. Another miRNA implicated in beta cell dysfunction is miR-29, which inhibits glucose-induced insulin secretion when increased in both mouse and human pancreatic islets [54]. Furthermore, recent studies indicated that seven miRNAs showed decreased plasma levels in diabetic mice compared to normoglycemic counterparts (miR-126a-3p, miR-126a-5p, miR-155, miR188-3p, miR-204, miR-218, and miR-409-3p) and using single-assay Real-Time PCR, five out of these miRNAs were validated as differentially expressed (miR-126a-3p, miR-126a-5p, miR-155, miR-204 and miR-409-3p [57].

Specifically, miR-409-3p was discovered to exhibit decreased expression levels in immune islet infiltrates of diabetic mice as a function of insulitis severity. Notably, miR-409-3p was found to be enriched in CD8+ central memory T cells. The plasma concentrations of this microRNA gradually declined during diabetes progression in mice and showed improvement upon illness remission following anti-CD3 antibody therapy [57].

In human plasma samples, lower levels of miR-409-3p were observed in individuals recently diagnosed with T1D compared to controls, with levels showing an inverse correlation with circulating HbA1c [57]. Additionally, miR-155, miR-92a, and miR-126 were significantly decreased in T1D serum samples [58]. miR-92a appears to play a role in regulating NF-κB and other inflammatory pathways, potentially contributing to the progression of cardiovascular disease associated with diabetes [59]. Similarly, miR-126 is crucial for maintaining endothelial homeostasis, a characteristic often disturbed in diabetes [60]. Furthermore, miR-126 regulates endothelial inflammation in individuals with micro/macrovascular complications linked to diabetes, establishing a link between diabetes-related complications and reduced levels of miR-126 [61].

Various research groups have found that miR-375 is upregulated alongside other miRNA molecules like miR-200 and miR-7 [62,63]. Barutta [58] and colleagues conducted a cross-sectional nested case-control study to investigate the varying expression of miRNAs in blood samples from individuals with T1D. They observed significant upregulation of miRNAs including miR-140-3p, miR-574-3p, miR-139-5p, miR-106a, miR-17, miR-486-3p, miR-16, miR-222, and miR-885-5p. Moreover, children with newly diagnosed T1D exhibited notably elevated levels of miR-197 in their blood samples, suggesting that miR-197 could accurately predict residual beta cell function [64].

Among microRNAs analyzed in multiple studies, approximately 21 (miR-15b, miR-20b-5p, miR-22-3p, miR-21-5p, miR-25-3p, miR-24-3p, miR-26b-5p, miR-27b-3p, miR-100-5p, miR-148a-3p, miR-146a-5p, miR-181a-5p, miR-150-5p, miR-200c-3p, miR-210-5p, miR-335-5p, miR-375, miR-342-3p, miR-1275, let-7g-5p, and let-7f-5p) were detected in plasma/serum or T/peripheral blood mononuclear cell (PBMCs)/cells, showing promise as circulating biomarkers for T1D [65].

However, only 11 out of these miRNAs consistently displayed dysregulation across multiple studies, having been examined in the same tissue by at least two investigations: miR-146a-5p, miR-150-5p, miR-342-3p, and miR-1275 resulted downregulated in PBMCs from individuals with T1D compared to controls, while miR-21-5p, miR-24-3p, miR-100-5p, miR-148a-3p, miR-181a-5p, miR-210-5p, and miR-375 were upregulated [65]. Furthermore, two studies explored miRNA expression profiles in the pancreas of murine models of T1D, revealing that miR-26a-5p expression was reduced in the pancreas of mice with either non-obese diabetic or streptozotocin-induced diabetes in comparison to control mice [66,67]. Nonetheless, to date, no investigation has explored miRNA expressions in the human pancreas in patients with T1D and in non-diabetic controls.

Moreover, miR-151-3p, let-7a-5p, and let-7c-5p exhibited downregulation in the pancreas of diabetic mice and PBMCs/T cells from individuals with T1D when compared to their respective control groups. Conversely, several miRNAs were downregulated in the pancreas of diabetic mice but upregulated in PBMCs or serum/plasma from T1D patients, indicating potential differences in expression patterns across tissues and/or species [66,67]. Upregulated miRNAs play a role in controlling inflammation by targeting genes involved in cytokine and immune cell activation pathways.

Elevated levels of miR-146a are observed in T1D, where it targets inflammation-related genes like tumor necrosis factor receptor-associated factor 6 (TRAF6) and interleukin-1 receptor-associated kinase 1 (IRAK1), thereby amplifying the inflammatory response. This could potentially lead to beta cell death. Additionally, miR-146a may also target genes crucial for beta cell function and survival [68,69]. Similarly, in the context of T1D, miR-375 shows increased levels. This miRNA inhibits genes responsible for insulin production, consequently disrupting glucose regulation [69].

It is also necessary to take into consideration that research on circulating miRNAs in T1D has primarily focused on newly diagnosed pediatric patients [70,71,72,73]. However, studying diverse populations, including those with long-standing diabetes, could offer valuable insights into disease progression. Factors such as weight and body composition also impact prognosis [74,75]. A recent study [76] has evaluated miRNA expression profiles in T1D without other pathologies, and it was observed that the development of a well-established disease, where patients are continuously treated with insulin, may differ from the onset of the disease and, more importantly, from other types of diabetes. Differences in miRNA expression may correspond to different disease stages. For instance, miR-1-3p in plasma, along with glycaemic control, could serve as a prognostic biomarker, potentially preventing vascular complications.

While studies like these hint at the potential of microRNAs for monitoring therapeutic interventions in T1D, their application in individuals at risk for the disease remains largely unexplored. Therefore, much more research is needed to fully validate these molecules as reliable tools for prediction or prognosis in T1D.

Further analysis is needed to understand the scope of miRNA alterations and their relationship with clinical complications. Long-term monitoring of patients will elucidate the development of any comorbidities. The exploration of miRNAs as potential biomarkers for T1D progression and therapeutic responsiveness could signify a promising avenue for personalized medicine.

**Figure 2:**
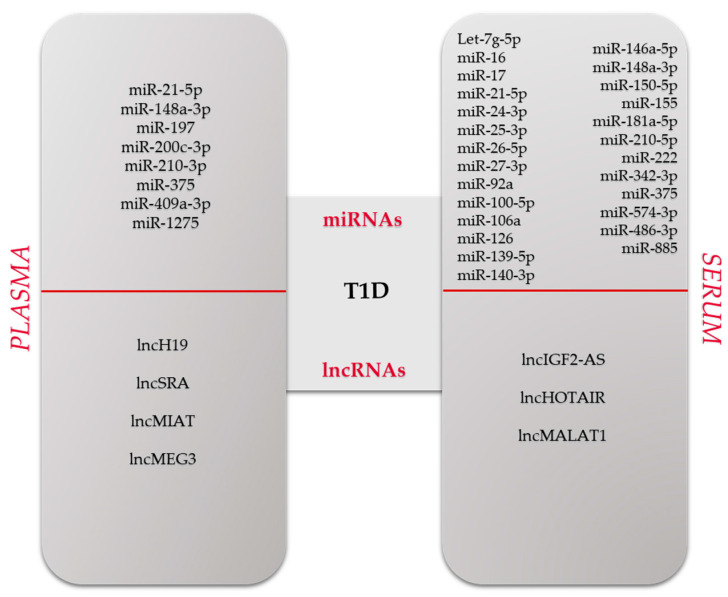
(**top panel**) miRNAs. (**bottom panel**) lncRNAs expressed in human plasma/serum of T1D patients.

Figure 2(top panel) summarizes the miRNAs expressed in human plasma/serum of T1D patients.

### 1.2. Long Non-Coding RNA and T1D

Long non-coding RNA plays a crucial role in regulating various physiological functions such as epigenetic modifications, transcriptional and post-transcriptional regulation, as well as the processing of small ncRNAs during cellular development and homeostasis [77]. Numerous in vitro and in vivo studies highlighted the potential involvement of lncRNAs in the pathogenesis of diabetes. These include processes such as pancreatic beta cell function and insulin secretion, as evidenced by lncRNAs like Insulin-Like Growth Factor 2 Antisense (IGF2-AS), βlinc1, and Maternally Expressed 3 (MEG3) [78,79,80,81,82]. Additionally, lncRNAs like H19 and steroid receptor RNA activator (SRA) have been implicated in regulating glucose homeostasis and insulin sensitivity [83,84]. Initially identified as transcriptional coactivators, lncRNA SRAs were found to participate in glucose homeostasis and AKT-dependent insulin sensitivity in adipocytes [85,86].

A study [87] revealed the upregulation of lncRNA SRA in plasma samples from 25 subjects diagnosed with T1D, as well as in CD4+ regulatory T cells (Tregs) exposed to high glucose in the CD4+ MOLT4 human T lymphoblast cell line. LncRNA SRA modulates the miR-146b expression, which is downstream of the interleukin-1 receptor-associated kinase 1 (IRAK1), AKT, and S6 Kinase 1 (S6K1) signaling pathways. Interestingly, lncRNA SRA and miR-146b engage in reciprocal regulation, suggesting that lncRNA SRA interacts with IRAK1/AKT/mTOR signaling by competing with miR-146b. Additionally, a positive correlation was observed between plasma lactate levels and HbA1c in T1D patients. LncRNA SRA plays a significant role in T1D by inhibiting miR-146b, thereby promoting the IRAK1/lactate dehydrogenase A (LDHA)/phosphorylated LDHA (pLDHA) signaling pathway. Another noteworthy lncRNA, MALAT1, has attracted attention due to its diverse roles in various physio-pathological processes [88,89,90,91,92]. MALAT1 is involved in the development of many autoimmune and inflammatory conditions, including T1D, due to its regulatory functions in immune cell differentiation, cytokine production, and inflammatory pathways. This suggests its potential as a multifaceted therapeutic target across a spectrum of multifaceted diseases [93,94,95].

Ding et al. [96] explored the role of MALAT1 in regulating cellular activity in T1D, demonstrating that MALAT1 contributes to cellular dysfunction and inhibits insulin production by reducing H3 histone acetylation at the PDX-1 promoter.

Diabetic nephropathy, or diabetic kidney disease (DKD), is a severe complication of both T1D and T2D. The role of TUG1 and MALAT1 was investigated in contributing to DKD in T1D patients, and while there was no difference in TUG1 expression between the groups, patients with T1D and DKD exhibited higher urinary MALAT1 expression than those without DKD [97].

Wong et al. established biomarkers for islet quality, which could be utilized before isolating islets for cell therapy in T1D. Their analyses identified two frequently observed MALAT1 variations. Validation using a set of 75 human islet preparations confirmed these results. The findings suggested that evaluating MALAT1 variant levels alone provides excellent specificity in predicting post-isolation islet quality. When combined with other selection approaches, it enhances the predictive potential for clinical islet transplantation. The biomarker MALAT1 represented a significant improvement over previously employed selection methods for islet isolation at the clinical level [98]. Consistent dysregulation of MALAT1 is associated with disease progression and severity. Understanding the diverse functions of MALAT1 suggests its potential as a therapeutic target. Various strategies for targeting MALAT1 show promise in modulating abnormal immune responses and potentially alleviating disease symptoms. However, further investigations are needed to ensure safety and effectiveness in clinical contexts. A precise understanding of how MALAT1 influences immune responses could pave the way for targeted therapies. Drugs regulating the expression of MALAT1, or its downstream effects, may offer more effective and precise treatments. Integrating MALAT1 data into personalized medicine approaches could lead to tailored treatments for individual patients based on their genetic and molecular profiles, potentially improving therapeutic outcomes. Collaborative efforts among researchers from different disciplines, including molecular biology, immunology, and bioinformatics, are essential to fully unravel the complexities of MALAT1 in autoimmune diseases. Additionally, exploring the interaction between MALAT1 and other ncRNAs, as well as their interactions with coding genes, could provide a more comprehensive understanding of the molecular mechanisms underlying autoimmune pathogenesis.

Maternally Expressed Gene 3 (MEG3) is a lncRNA found in various isoforms situated on chromosome 14q32.2 in humans. Its primary function involves the regulation of apoptosis, and its involvement in the MAPK, PI3K/Akt, and VEGF signaling pathways has been noted [99]. The expression of MEG3 plays a crucial role in insulin production and secretion from pancreatic beta cells. In BALB/c mice, MEG3 expression levels were elevated in pancreatic islets but decreased in models of both Type 1 and Type 2 diabetes. Research indicates that glucose levels regulate MEG3 expression in pancreatic beta cell lines and murine cells [82]. Knockdown of MEG3 has been associated with diminished insulin secretion and increased beta cell apoptosis, mediated by decreased levels of two vital transcription factors necessary for insulin expression in beta cells [100,101].

As also reported above, there is increasing evidence suggesting that EVs, specifically exosomes, play a significant role in various pathogenic processes, including T1D [31,32,102,103,104,105,106,107,108,109]. Moreover, the content of exosomes is carefully controlled in response to both internal and external signals, thus reflecting biological processes or disease states [110]. Therefore, delving deeper into exosomes could provide dear insights into the diagnosis and treatment of diseases.

Although increasing evidence has emphasized the role of lncRNAs in pancreatic islets and the pathogenesis of T1D [110,111,112,113,114], research on exosomal lncRNAs remains relatively limited. In a recent study [115], a comprehensive analysis of plasma-derived exosomal lncRNA expression profiles in T1D was conducted, revealing 162 dysregulated exosomal lncRNAs, with 77 showing upregulation and 85 showing downregulation.

Among these, the Long intergenic non-coding RNAs (lincRNAs) accounted for the highest proportion of differentially expressed lncRNAs, which agrees with a previous study [116]. LincRNAs are characterized as autonomously transcribed ncRNAs that have longer than 200 nucleotides and that do not overlap with annotated coding genes. They share characteristics with other lncRNA transcripts and represent over half of the lncRNA transcripts in humans. The existence of lincRNAs was initially proposed through studies utilizing tiling arrays across genomic sequences, which revealed pervasive transcription [117,118] from regions with no known coding genes [119,120,121,122]. Early support for active transcription units at these putative loci was provided by assessing chromatin state signatures in murine cell types [123]. LincRNAs have been differentiated from the broader category of lncRNA transcripts, as many lncRNAs share sequences with coding loci. However, in many publications, these two sets of transcripts are not distinguished, and they are collectively referred to as “lncRNAs” [116].

Figure 2 (bottom panel) reports the lncRNAs expressed in human plasma/serum of T1D patients.

### 1.3. Circular RNA and T1D

Circular RNAs (circRNAs) were identified more than 20 years ago [124] and were considered the result of rare splicing errors. They are characterized by their unique structure, forming a covalently closed continuous loop due to the joining of the 3′ and 5′ ends typically found in RNA molecules. Their resistance to exonuclease-mediated degradation confers exceptional stability compared to other ncRNAs within cells [125]. CircRNAs exhibit diverse functionalities; for example, they act as miRNA sponges, they compete in mRNA splicing, and they participate in the transcription or post-transcriptional regulation of target genes, but most circRNAs do not encode for protein [126,127]. Abundant in various body fluids, such as peripheral blood, saliva, urine, and semen, circRNAs have emerged as promising candidates for disease research owing to their stability and abundance. Little is known, however, about their functional role [128,129].

According to a recent review [130], numerous investigations made an effort to discover novel circRNAs and their possible biological roles in autoimmune diseases, given the widespread reporting of abnormal expression levels of numerous circRNAs in these diseases. Remarkably, circRNAs not only serve as diagnostic molecular markers but also play pivotal roles as regulators or therapeutic targets at disease onset and during its progression. Consequently, research into circRNA functions within immune cells is rapidly expanding, with established roles in modulating immune responses.

In a study by Stoll et al. [131], it was found that CiRS and circHIPK3 contributed to the regulation of essential beta cell activities and displayed altered expression in diabetes models, suggesting that they may be implicated in the development of this disease.

Subsequently, thirty upregulated and sixty-three downregulated circRNAs were identified in the peripheral blood of T1D patients, among which there were circRNAs (hsa_circ_0002473 and hsa_circ_0072697) [132]. Additionally, GO and KEGG analyses associated these circRNAs primarily with non-homologous end-joining, RIG-I-like receptor signaling pathway, NF-κB signaling pathway, and cell cycle, all linked to T1D development [132]. Another study by Li et al. found significant upregulation of four circRNAs (hsa_circRNA_101062, hsa_circRNA_100332, hsa_circRNA_085129, and hsa_circRNA_103845) in the plasma of newly-onset T1D patients [133]. Notably, circRNAs regulate gene expression by interacting with miRNAs. Pang et al. reported hsa_circ_0005630-miR-1247-5p-ATXN1/ARL6IP1 and hsa_circ0007026-miR-324-5p-NCAPD2/PGAM1 as potentially involved in T1D progression [134]. These findings strongly suggest that dysregulated circRNAs are associated with T1D progression and may serve as biomarkers for diagnosis. Some circRNAs contribute to the inflammatory response and signaling pathways in T1D. For instance, circPPM1F, predominantly found in monocytes and upregulated in individuals with T1D, activates M1 macrophages through the circPPM1F-HuR-PPM1F–NF-κB pathway [135]. As early macrophage levels surge in early T1D stages, inhibiting circPPM1F may hold promise for early T1D treatment [136]. Similarly, as other circRNAs act as miRNA sponges [137], it is suggested that Cdr1as (CiRS-7) sponge miR-7 can regulate insulin transcription and secretion in islet cells [138]. Additionally, hsa_circ_0060450, upregulated in peripheral blood mononuclear cells, acts as a miR-199a-5p sponge, suppressing the JAK-STAT signaling pathway induced by IFN-I, thus dampening macrophage-mediated inflammatory response [139]. Overall, circRNAs appear to exert regulatory roles in T1D, but additional investigations are warranted to understand their functional implications in T1D treatment comprehensively. Enhanced comprehension of the functions of these recently unearthed RNA variants holds the potential to illuminate the mechanisms contributing to beta cell dysfunction in diabetic contexts. Furthermore, it may pave the way for the identification of innovative approaches to prevent or manage this prevalent metabolic disorder.

## 2. Conclusions

Given the uncertainty surrounding the precise origins of T1D, the detection of new biomarkers and their role in the pathogenesis of this condition could significantly improve our understanding of its underlying mechanisms. From a clinical perspective, these new biomarkers may have the potential to facilitate early diagnosis and more personalized treatment approaches for T1D patients, thereby improving their overall quality of life. Circulating miRNAs emerge as promising biomarkers due to their stability, resilience to degradation by ribonucleases or freeze–thaw cycles, and detectability in body fluids through highly sensitive and specific quantitative methods.

However, it is important to highlight that not all measured miRNA expressions necessarily reflect biological activity. In fact, only measurements of miRNAs incorporated within the RISC complex really indicate that they are biologically active. In addition, ncRNAs (at least miRNAs) are very abundant and usually expressed in several tissues and cell types where they might have very different functions. These represent one major drawback of miRNAs as potential therapies since targeting them systemically is most likely problematic.

Although further studies are necessary to better understand their role in T1D, the identification of new ncRNAs could also play an important role and prove to be promising biomarkers in diagnosis and therapeutic development.

Through the development of personalized medicines and the adoption of new diagnostic and prognostic techniques, healthcare professionals can improve their understanding of diseases and conduct personalized clinical assessments for each patient.
